# Highly Virulent Newcastle Disease Virus in Eurasian Collared Doves in the North of Portugal

**DOI:** 10.3390/ani15243563

**Published:** 2025-12-11

**Authors:** Guilherme Moreira, Soraia Rodrigues, Sara Gomes-Gonçalves, Gabriela Silva, Irina Amorim, Eliane Silva, Sofia Carmezim, Vanessa Soeiro, João R. Mesquita

**Affiliations:** 1 Clínicas Veterinárias, Instituto de Ciências Biomédicas Abel Salazar (ICBAS), Universidade do Porto, 4050-313 Porto, Portugal; 2 Institute for Research and Innovation in Health (i3S), University of Porto, 4200-135 Porto, Portugal; 3 Institute of Molecular Pathology and Immunology (IPATIMUP), University of Porto, 4200-135 Porto, Portugal; 4 Research Center in Biodiversity and Genetic Resources (CIBIO/InBIO), University of Porto, 4485-661 Porto, Portugal; 5 Wildlife Rehabilitation Center of Parque Biológico de Gaia (CRF-PBG), 4430-812 Vila Nova de Gaia, Portugal; 6 Centro de Estudos de Ciência Animal (CECA), Instituto de Ciências, Tecnologias e Agroambiente (ICETA), University of Porto, 4051-401 Porto, Portugal; 7 Associate Laboratory for Animal and Veterinary Science (AL4AnimalS), 4050-313 Porto, Portugal

**Keywords:** avian orthoavulavirus, Columbidae, wildlife, metagenomics, Portugal, Newcastle disease virus, velogenic pathotype

## Abstract

Newcastle disease (ND), caused by avian orthoavulavirus 1 (AOAV-1), poses a global threat to poultry and wild birds. In early 2025, an outbreak of pigeon paramyxovirus type 1 (PPMV-1, genotype VI AOAV-1) was detected in a wildlife rehabilitation centre in northern Portugal, affecting *Streptopelia decaocto*, *Streptopelia risoria*, and *Columba livia*. Birds showed acute neurological signs and died rapidly. Necropsy revealed brain and pulmonary congestion, splenomegaly, and cloacal lesions, while histopathology demonstrated hepatocellular necrosis, hemorrhage, and eosinophilic intracytoplasmic inclusions in hepatocytes and renal tubular cells. Matrix (M) gene PCR using standard primers was negative, but metagenomic sequencing identified genotype VI as being closely related to strains from Iran and Cyprus. Partial fusion (F) gene analysis revealed the velogenic RRQKRF motif. These findings confirm the circulation of highly virulent PPMV-1 in Portugal, highlight that standard, recommended primers may fail to detect some genetically diverse strains, and emphasize the role of Columbidae as reservoirs with potential transmission to domestic poultry.

## Simple Summary

Newcastle disease is a contagious viral infection that affects poultry and wild birds. In early 2025, a severe outbreak of pigeon paramyxovirus type 1 (PPMV-1) occurred in a wildlife rehabilitation centre in northern Portugal. The virus caused sudden nervous signs and death in pigeons and doves. Routine diagnostic tests failed to detect the infection, but genetic sequencing confirmed the presence of a highly virulent virus closely related to strains from the Middle East and Cyprus. These findings show that pigeons and doves can act as important carriers of dangerous Newcastle disease viruses, which may spread to domestic poultry.

## 1. Introduction

Newcastle disease (ND) is a highly contagious and economically significant viral disease, with particular impact on domestic poultry, and the World Organization for Animal Health (WOAH) defines ND as an infection with *Avian paramyxovirus type 1* (APMV-1) specifically in poultry [1].

The causative agent, avian orthoavulavirus 1 (AOAV-1), commonly named Newcastle disease virus (NDV), is an enveloped, pleomorphic (but usually spherical), negative-sense, non-segmented member of the Paramyxoviridae family [2]. Its genome comprises around 15 kilobases, with 6 to 8 open reading frames encoding 6 to 8 proteins: NP (nucleoprotein), P (phosphoprotein), M (matrix protein), F (fusion protein), L (large polymerase), and HN (hemagglutinin–neuraminidase) [3]. First identified in Java and later in Newcastle-upon-Tyne in 1926, it is classified under the genus *Orthoavulavirus*, in the sub-family Avulavirinae, belonging to the family Paramyxoviridae [4]. Infections have been reported in over 241 bird species across 27 orders, indicating a broad host range. Clinical signs vary significantly depending on the bird species and the viral strain involved. The virions usually enter the host organism through the respiratory or gastrointestinal epithelial cells [5,6,7]. AOAV-1 is classified into two major classes. Class I contains a single genotype, is primarily detected in wild waterfowl, is generally avirulent, and exhibits lower genetic diversity. Class II comprises at least 21 genotypes (I–XXI, with additional sub-genotypes), occurs in both poultry and wild birds, and includes strains ranging from low to high virulence, being responsible for most domestic poultry outbreaks [7,8]. Within Class II, genotypes I and II encompass both low- and high-virulence strains, with genotype II commonly used in vaccines; genotypes V, VI, and VII are highly virulent, widely distributed, and implicated in recent global epidemics, and characterized by high potential for interspecies spread, including the possibility of reintroduction of viruses into domestic poultry, whereas other genotypes (XI, XIII, XIV, XVI, XVII, XVIII, XXI) tend to be geographically restricted and often associated with specific regions or host species [7,8]. AOAV-1 was originally divided into pathotypes: lentogenic (avirulent), mesogenic (moderate virulence), and velogenic. The WHOA defines virulent strains according to the intracerebral pathogenicity index (defined as the mean score of birds over an eight-day period, where 0 is healthy, 1 is sick, and 2 is dead). Highly virulent strains approach a score of 2, where most birds die a day after inoculation [1]. AOAV-1 was originally divided into pathotypes: lentogenic (avirulent), mesogenic (moderate virulence), and velogenic. The WHOA defines virulent strains according to the intracerebral pathogenicity index (defined as the mean score of birds over an eight-day period, where 0 is healthy, 1 is sick, and 2 is dead). Highly virulent strains approach a score of 2, where most birds die a day after inoculation [1].

Outbreaks of AOAV-1 have been reported in several parts of the world from different avian species, representing a significant threat to the poultry industry. It is now considered endemic in most areas globally, with high potential for significant economic losses [8].

Pigeon paramyxovirus type 1 (PPMV-1) is an antigenic variant of AOAV-1, constituting Group VI, which is mainly associated with infections of pigeons and has the potential to result in disease in chickens [9,10]. Pigeons of all ages are susceptible to PPMV-1 infection, which has been associated with a high rate of morbidity and mortality in pigeons [10]. PPMV-1 was initially isolated in the Middle East in the 1970s and rapidly spread throughout numerous countries, leading to tremendous economic losses [11].

This study aimed to investigate the cause of sudden mortality among Columbidae in a wildlife rehabilitation centre in northern Portugal, using clinical, pathological, and molecular methods.

## 2. Materials and Methods

### 2.1. Sampling

Between 25 January and 3 March 2025, a high number of Eurasian collared doves (*Streptopelia decaocto*, *n* = 11) were admitted to a Wildlife Rehabilitation Centre in northern Portugal. The birds presented with a range of clinical signs of varying severity, from lethargy and anorexia to pronounced neurological manifestations, including ataxia, head tilting (torticollis), tremors, and loss of coordination. All affected doves developed severe and progressive neurological signs, ultimately dying within 24 h of onset.

**Figure 1:**
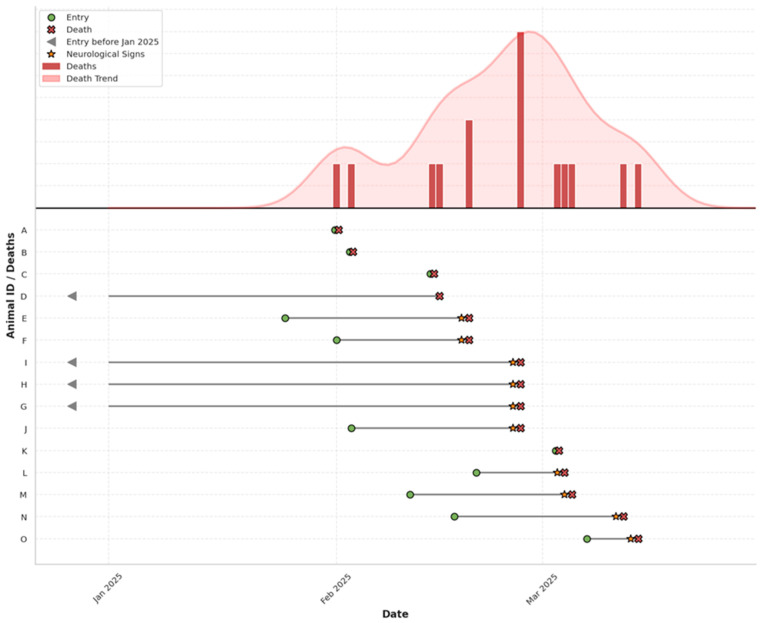
Epidemic curve (**above**) and timeline (**below**) of PPMV-1 outbreak in Columbidae at a wildlife rehabilitation centre. Individual-level timelines for affected birds (A–O) are plotted, indicating entry, onset of neurological signs, and death. Arrows denote individuals present before January 2025.

During the same period, similar neurological signs were observed in three Barbary doves (*Streptopelia risoria*) and one fancy domestic pigeon (*Columba livia f. domestica*, fan-tailed variety) permanently housed at the park. These birds exhibited comparable clinical progression and also died within 24 h of the onset of neurological signs (Figure 1).

The temporal clustering, rapid neurological deterioration, high mortality rate, and involvement of the central nervous system raised strong suspicion of an AOAV-1 outbreak.

All Columbidae specimens were subjected to a complete necropsy protocol, during which major organs were examined and any macroscopic lesions or abnormalities were documented. Representative liver and kidney samples from all individuals were collected. For histopathological evaluation, tissues were fixed in 10% neutral buffered formalin, routinely processed, and embedded in paraffin. Sections 2 μm thick were cut and stained with hematoxylin and eosin (H&E) for microscopic visualization. Slides were then examined using a light microscope (Nikon Eclipse 50i (Nikon Corporation, Tokyo, Japan)). Due to advanced autolysis, none of the collected brain tissue samples were suitable for histopathological evaluation. From the available specimens, a single individual was selected for genomic sequencing based on the relative freshness of the carcass. Organ samples from this individual were promptly preserved at −80 °C to prevent nucleic acid degradation and stored until further processing. All remaining biological material from the specimens was safely disposed of following standard biohazard protocols.

### 2.2. Nucleic Acid Extraction

Total RNA was extracted from approximately 20–30 mg of bird brain tissue using the QIAamp Viral RNA Mini Kit (Qiagen, Hilden, Germany) and the QIAcube^®^ automated platform (Qiagen, Hilden, Germany), with protocol modifications to include mechanical homogenization and proteinase K digestion. Tissue samples were first homogenized using a TissueLyser bead mill (Qiagen) in the presence of approximately 400 μL of AVL buffer (Qiagen), pre-mixed with carrier RNA, and 25 μL of proteinase K. Samples were incubated at 37 °C for 10 min to facilitate lysis and protein digestion. After incubation, AVL buffer was added to bring the final volume to 700 μL, followed by brief centrifugation to remove debris.

The clarified lysate was then transferred to the QIAcube instrument, and total RNA was extracted using the Viral RNA Mini Kit protocol with off-board lysis. RNA was eluted in 60 μL of RNase-free water and stored at −80 °C until further use.

### 2.3. PCR Amplification

PCR assays targeting the matrix protein (*M*) gene using the primer pair M-4100 (5′-AGTGATGTGCTCGGACCTTC-3′) and M-4220 (5′-CCTGAGGAGAGGCATTTG CTA-3′) [12].

All PCR reactions were run on a T100 thermocycler (Bio-Rad, Hercules, CA, USA). Reaction mixtures were performed using the Xpert One-Step RT-PCR kit (GRiSP^®^, Porto, Portugal), in accordance with the manufacturer’s instructions. Conditions included cDNA synthesis at 45 °C for 15 min, initial denaturation at 95 °C for 3 min, 40 cycles of denaturation at 95 °C for 15 s, annealing at 52 °C for 15 s, extension at 72 °C for 2 s, and final extension at 72 °C for 10 min. The amplified DNA fragments were identified by electrophoresis on 1.5% agarose gels, stained with Xpert Green Safe DNA gel dye (GRiSP^®^, Porto, Portugal), at 100 V for 30 min. UV light irradiation was used to visualize the results.

### 2.4. Sequence-Independent Single-Primer Amplification

A previously described SISPA protocol [13,14] was followed with minor modifications, as outlined below.

Following RNA extraction, samples were treated with RNase-free DNase I (Qiagen) according to the manufacturer’s instructions to eliminate residual genomic DNA. The DNase-treated RNA was then used as input for sequence-independent single-primer amplification (SISPA) to enable unbiased reverse transcription and amplification of RNA-derived sequences.

First-strand cDNA synthesis was performed using SuperScript IV reverse transcriptase (Thermo Fisher Scientific, Waltham, MA, USA) and a primer composed of a known anchor sequence fused to a random nonamer (5′-GTTTCCCACTGGAGGATA-N_9_-3′). Reactions were incubated at 25 °C for 10 min, followed by 50 °C for 50 min, and inactivated at 70 °C for 15 min.

Second-strand synthesis was conducted using Sequenase Version 2.0 DNA Polymerase (Thermo Fisher Scientific) with a two-step protocol in which Sequenase was first added to the first-strand reaction and incubated at 37 °C for 10 min, followed by the addition of a second aliquot of Sequenase and an additional 10 min incubation at 37 °C to ensure complete synthesis of the complementary strand, without purification between cDNA synthesis steps. The resulting double-stranded cDNA was then amplified by PCR using a primer corresponding to the known anchor sequence (5′-GTTTCCCACTGGAGGATA-3′) and Q5 High-Fidelity DNA Polymerase (New England Biolabs, Ipswich, MA, USA) under the following thermal cycling conditions: 98 °C for 30 s; 35 cycles of 98 °C for 10 s, 55 °C for 20 s, and 72 °C for 30 s; followed by a final extension at 72 °C for 2 min.

### 2.5. Metagenomic Sequencing

SISPA-prepared cDNA was sequenced using Oxford Nanopore Technology (ONT) via a PromethION 24 instrument (Oxford Nanopore Technologies, Oxford, England, UK), equipped with a R10.4.1 flow cell. The library preparation utilized the Native Barcoding Kit 96 V14 (SQK-NBD114.96) (Oxford Nanopore Technologies, Oxford, England, UK). The raw FASTQ reads were basecalled in super-accurate mode, using ont-doradod-for-promethion v.7.4.12, applying a minimum Q-score of 10, with adapters and barcodes trimmed via MinKNOW (Oxford Nanopore Technologies, Oxford, England, UK). Initial quality control of the sequencing reads was performed using NanoPlot 1.43.0 [15]. Sequencing adapters and barcodes were removed using Porechop 0.2.4 [16]. To filter the reads, NanoFilt v.2.8.0 [15] was employed, applying a minimum average quality threshold of 10.

### 2.6. Pathogen Identification

Host and contaminant-associated reads were removed by aligning the raw sequencing reads to reference FASTA files corresponding to the host and potential contaminants using Minimap2 (v2.28-r1209) [17]. Reads that did not align to these sequences (i.e., unmapped reads) were retained for downstream analysis. These unmapped reads were taxonomically classified using Kraken2 (v2.1.3) [18] against the RefSeq viral database to identify putative viral sequences. Reads assigned to *Avian orthoavulavirus 1* were subsequently extracted using the extract_kraken_reads.py (v3.1) [19] script, which selectively retrieves reads based on Kraken2 classification output.

The extracted reads were then aligned to a reference genome of *Avian orthoavulavirus 1* using a custom Python (v3.8.15) script developed in-house [20]. Briefly, the reference genome was first indexed using Minimap2 (version 2.30) [17], a fast and accurate aligner optimized for long-read data such as that generated by Oxford Nanopore Technologies. The reads were aligned using Minimap2 with the map-ont preset, producing a SAM file, which was subsequently converted to a sorted BAM format and indexed using Samtools [21] (version 1.22) to facilitate efficient data handling. Coverage depth across the genome was calculated from the sorted BAM file using Pysam (version 0.23.3) [22], a Python interface for the SAM/BAM format. Per-base coverage was obtained by iterating through pileup columns, and the resulting coverage profile was visualized using Matplotlib (v.3.10) [23].

Finally, a consensus sequence was generated from the aligned reads based on regions of sufficient coverage and read overlap. This consensus sequence was then queried against the NCBI nucleotide database using nucleotide BLAST v.2.17.0 [24] to confirm viral identity and assess sequence similarity.

### 2.7. Phylogenetic Analysis

Two phylogenetic analyses were conducted to better resolve taxonomic placement.

Firstly, a phylogenetic tree was constructed to provide broad taxonomic context across the *Paramyxoviridae* family. This low-resolution tree included representatives from all recognized genera and subfamilies, using available full genome sequences trimmed to match the region covered by our consensus sequence, specifically the *matrix (M)* gene and its flanking regions. Sequence alignment was performed using MAFFT (v.7.525) [25], and a maximum likelihood tree was inferred using IQ-TREE (v3.0.1) [26] with automatic model selection and branch support assessed through 1000 bootstrap replicates. To achieve higher phylogenetic resolution, a second tree was constructed using a more focused dataset. This analysis included our consensus sequence, the top nucleotide BLAST [24] (GenBank, retrieved on 5 June 2025) hits, and a curated set of full genome sequences representing diverse strains, classes, and genotypic groups within *Avian orthoavulavirus 1*. All sequences were trimmed to the *M* gene and partial *F* gene regions to allow homologous comparison. This approach enabled a more detailed investigation of the sequence’s relationship within the known diversity of AOAV-1. Alignment and phylogenetic inference were performed using the same methodology as described above. Both trees were visualized using the interactive tree of life [27] (ITOL V6) platform.

## 3. Results

### 3.1. Clinical Presentation and Disease Progression

During the study period, all examined birds exhibited a spectrum of clinical signs of differing intensity, ranging from lethargy and loss of appetite to prominent neurological symptoms such as ataxia, head tilt (torticollis), tremors, and impaired coordination. In all affected doves, the neurological signs were severe and rapidly progressive, leading to death within 24 h of symptom onset. Neurological signs were also observed in three Barbary doves (*Streptopelia risoria*) and one fancy domestic pigeon (*Columba livia f. domestica*, fan-tailed variety) permanently housed at the park. These birds exhibited comparable clinical progression and also died within 24 h of the onset of neurological signs (Figure 1).

### 3.2. Necropsy and Histopathology

All Columbidae specimens underwent complete necropsy, which consistently revealed brain congestion characterized by generalized vascular engorgement and a prominent vascular pattern. The lungs exhibited diffuse reddish discoloration, indicative of congestion or hemorrhage. Splenomegaly was observed in two birds, and two also presented with cloacal dilation, one of which had purulent exudate in the bursa of Fabricius. These findings are compatible with AOAV-1 infection.

**Figure 2:**
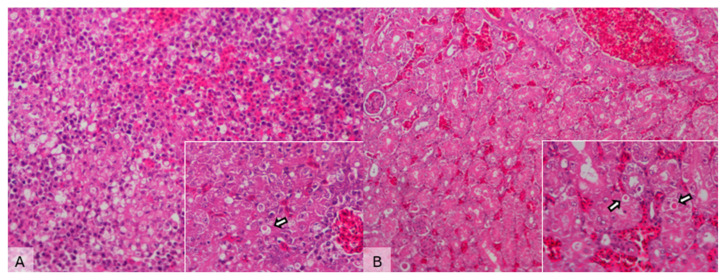
Microscopical images of liver and kidney sections from a *Streptopelia decaocto* specimen infected with Newcastle disease virus (NDV). (**A**) Liver—hydropic degeneration, multifocal hepatocellular necrosis, and hemorrhage (H&E, 200×). Inset: Note the presence of eosinophilic inclusion bodies (arrow) (H&E, 400×). (**B**) Kidney—mild tubular necrosis and vascular congestion (H&E, 100×). Inset: Eosinophilic inclusion bodies in the renal tubules (arrows) (H&E, 400×).

Histopathological examination showed multifocal hepatocellular necrosis and hemorrhage, occasionally accompanied by 2–5 μm acidophilic intracytoplasmic inclusions in hepatocytes and renal tubular cells, along with mild tubular necrosis. The spleen exhibited congestion, hemorrhage, and mild to moderate lymphoid depletion (Figure 2).

### 3.3. PCR

Conventional PCR assays targeting the matrix protein gene (*M* gene) using the primer pair M-4100 (5′-AGTGATGTGCTCGGACCTTC-3′) and M-4220 (5′-CCTGAG GAGAGGCATTTGCTA-3′) did not yield any detectable amplicons in the tested samples. No specific bands were observed in agarose gel electrophoresis, and amplification was not detected under the tested conditions.

### 3.4. Metagenomic Sequencing

**Figure 3:**
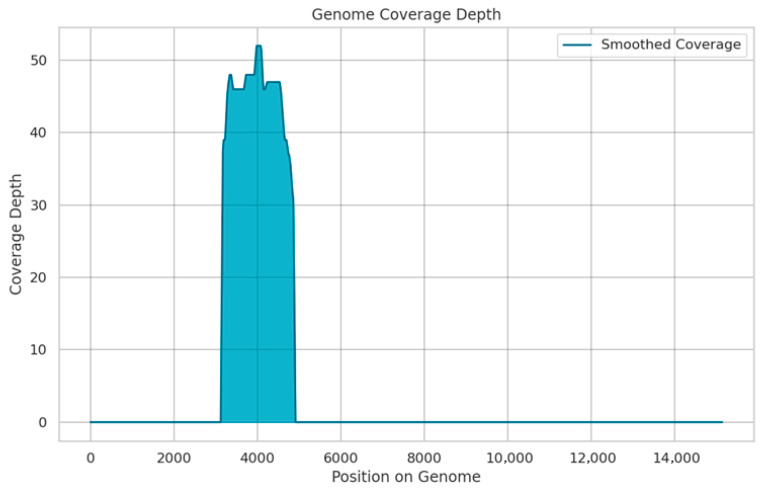
Coverage depth across the AOAV-1 genome detected by SISPA and Oxford Nanopore sequencing. The depth of coverage is shown on the y-axis, and the genome position is shown on the x-axis. (reference: MG456676).

Third-generation sequencing achieved complete coverage of the AOAV-1 Matrix (M) gene, spanning to 412 bp on the 5′ end of the gene. Of the 2388 reads retained after quality control, 56 (2.35%) successfully mapped to the reference genome (MG456676). The overall genome coverage was 11.42%, with an average sequencing depth of 45× across the covered regions (Figure 3).

The consensus sequence was confirmed by BLAST analysis against NCBI, showing the highest similarity (94.46%) to a PPMV-1 strain isolated from a collared dove in Iran in 2014 (PV137933), followed by a pigeon isolate from Cyprus (MG456676). These results indicate close genetic relatedness to Group VI AOAV-1 viruses circulating in Columbiformes. The consensus sequence has been deposited in GenBank under accession number PV763887.

**Figure 4:**
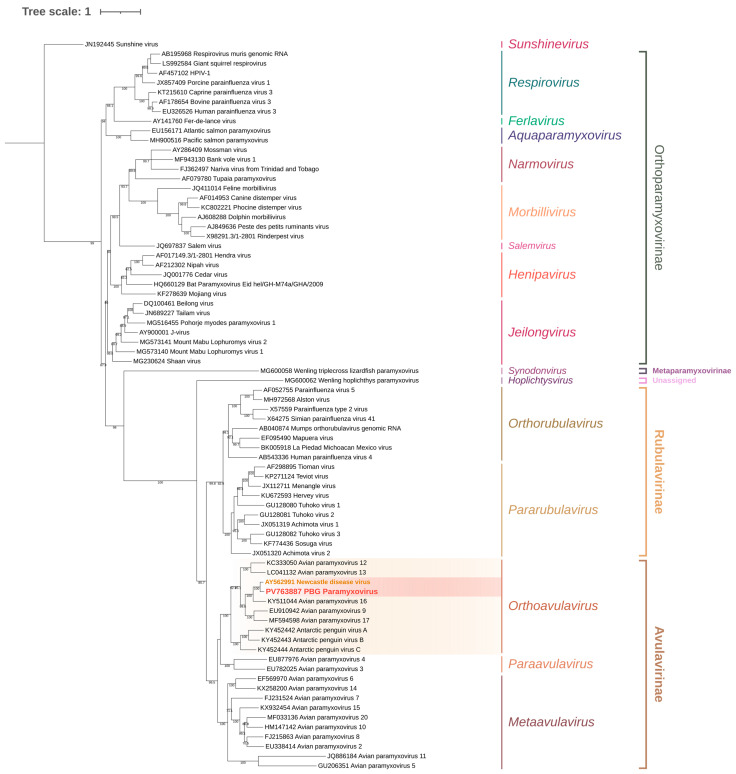
Maximum-likelihood phylogenetic tree of Paramyxoviridae based on the matrix (M) gene and adjacent incomplete F gene sequence. The tree was inferred using the TVM + F + R5 substitution model (Transversion Model with empirical base frequencies and a FreeRate model of rate heterogeneity using five discrete rate categories), selected as the best-fitting model according to the Bayesian Information Criterion (BIC). Sequences belonging to Orthoavulavirus are highlighted in yellow, while sequences grouped within the AOAV-1 node are highlighted in red. Branch support values are shown at the nodes where applicable.

**Figure 5:**
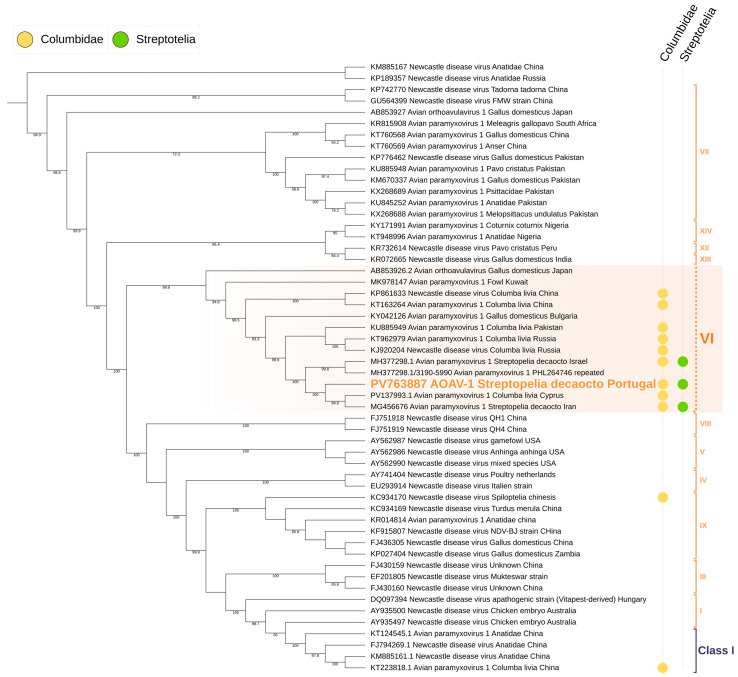
Maximum-likelihood phylogenetic tree of Paramyxiviridae based on the matrix (M) gene and incomplete fusion (F) gene regions. The tree was inferred using the TIMe + G4 substitution model (transition model with equal transversion rates and unequal transition rates, using a gamma distribution with four discrete categories to account for among-site rate heterogeneity), selected as the best-fit model according to the Bayesian Information Criterion (BIC). Sequences grouped within Group VI are highlighted in blue, sequences obtained from Columbidae are marked in yellow, and sequences obtained from *Streptotelia decaocto* are marked in green. Branch support values are shown at the nodes where applicable.

Phylogenetic analysis of the obtained sequences using maximum-likelihood methods confirmed that the virus clustered within the AOAV-1 Group VI clade, distinct from other Paramyxoviridae, confirming its identity as PPMV-1. Amino acid analysis of the 3′ fusion gene segment revealed the RRQKRF motif, characteristic of highly virulent velogenic strains (Figure 4 and Figure 5).

### 3.5. In Silico Primer Analysis

Primers and target sequences were aligned using Jalview (version 2.11.4.0) [28]. The primer sequences were manually input and aligned to the corresponding target DNA sequences. Alignment visualization enabled precise inspection of primer–target complementarity [29]. During the analysis, a single-nucleotide mismatch was identified between the primer and its complementary region in the target sequence at the 3′ end of the primer.

## 4. Discussion

Avian orthoavulavirus 1 (AOAV-1), the causative agent of Newcastle disease, is a significant pathogen affecting domestic poultry worldwide and has been reported in various wild bird species [8]. Despite its importance, data on AOAV-1 circulation in wild birds remain limited in many regions, including our study area. In this work, AOAV-1 Group VI (PPMV-1) sequences were detected in *Streptopelia decaocto* samples collected from the wild, with molecular analyses revealing close genetic similarity to strains previously reported in wild columbids from distant geographic locations.

Notably, the Portuguese isolate exhibited a high degree of phylogenetic relatedness to strains reported from Iran and Cyprus. This observation holds significant epidemiological relevance, as both countries are situated along major migratory bird flyways traversing the Mediterranean basin. Migratory avifauna may act as biological or mechanical vectors [30,31], facilitating viral dissemination across geographically distant regions through direct or indirect interactions with Columbidae populations. These phylogeographic connections support the hypothesis that the outbreak in Portugal is not an isolated occurrence but rather part of a broader network of viral circulation along these migratory routes.

International trade in pigeons and ornamental birds further amplifies the potential for viral dissemination. Commercial and recreational movements of avian species are frequent between Mediterranean and Middle Eastern countries, and the close genetic relatedness of the Portuguese isolate to Iranian and Cypriot strains raises the possibility of introduction via these trade pathways [32,33,34]. Iran and adjacent regions are recognized endemic foci of PPMV-1PPMV-1, representing plausible sources of variants capable of transcontinental spread into Europe. Elucidating these regional and intercontinental transmission networks is therefore critical for strengthening surveillance and implementing effective control strategies [34].

Standard PCR assays targeting the matrix (M) gene did not detect AOAV-1 in these samples, despite the use of the recommended primer set. This likely reflects sequence mismatches in genetically diverse strains, underscoring a known limitation in the primer design [35]. Consequently, surveillance strategies that rely exclusively on these M-gene primers may underestimate the true prevalence of AOAV-1. These findings highlight the value of metagenomic approaches for uncovering divergent or emerging variants. The detection of AOAV-1 in wild birds further emphasizes their role as reservoirs and vectors, contributing to the maintenance and potential spillover of the virus into poultry populations. Given the broad host range of AOAV-1 [8] and the ecological overlap between wild and domestic birds, comprehensive surveillance that includes wild bird populations is essential to capture viral diversity and improve disease prevention and control strategies.

Although partial *M* gene sequences provide useful information on AOAV-1 presence and relatedness, pathotypic classification relies on the fusion (*F*) gene. In our study, a partial *F* gene sequence was obtained containing the RRQKRF motif, characteristic of highly virulent velogenic strains and thus confirming the velogenic nature of the detected AOAV-1 [36,37,38]. Although we successfully amplified the M and partial F gene fragments, we were unable to recover the remainder of the viral genome, even using long-read nanopore sequencing. This limited coverage likely stems from a combination of factors, including a low viral load, degradation of genetic material in the original samples, and random inefficiencies during cDNA synthesis [39]. Under these challenging conditions, shorter, more stable genomic regions (like the structurally accessible M gene, which often persists in later infection stages) are more likely to be sequenced [40,41]. In contrast, longer or more complex genomic segments may remain undetectable. Therefore, the genomic data we obtained should be interpreted as a sign of which fragments are most stable, not as evidence that the rest of the genome was absent. This highlights the value of metagenomic approaches for identifying divergent viral strains, even when a complete genome cannot be assembled [42]. 

Gross necropsy consistently revealed brain and pulmonary congestion, splenomegaly, and cloacal lesions, while histopathology demonstrated multifocal hepatocellular necrosis, hemorrhage, and eosinophilic intracytoplasmic inclusions, providing pathological confirmation of infection consistent with velogenic AOAV-1. Regrettably, many collected samples were too degraded to yield reliable viral RNA for sequencing, limiting the number of specimens analyzed. Future work with better-preserved samples and larger cohorts is needed to clarify the virus’s pathogenic potential in wild birds.

AOAV-1 Group VI, otherwise known as Pigeon paramyxovirus 1 (PPMV-1), was first detected in 1981, isolated from pigeons suffering from “viral encephalomyelitis”. This variant was later recognized as the one responsible for the 3rd panzootic which spread across Europe in the early 1980s [43]. Subsequent research confirmed that PPMV-1 has been endemic in Columbiformes worldwide since its initial detection in the 1980s, causing numerous outbreaks in both pigeons and poultry [43]. The virus has since spread globally, with documented outbreaks and genetic diversification in regions including the British Isles, China, Iran, Australia, and North America [44]. There is some evidence pointing towards PPMV-1 originating from chicken AOAV-1, followed by host adaptation to columbiform species [45]. During sustained circulation within pigeon populations, virulence in Columbiformes increases with diminishing pathogenicity in Galliformes [46]. Necropsy and histopathological findings in *Streptopelia decaocto* support this notion, illustrating lesions compatible with AOAV-1 infection and underscoring the pathogenic impact of PPMV-1 in Columbidae. The potential for virulent AOAV-1 dissemination by pigeons and cormorants poses a longstanding risk to poultry populations [47,48]. Following infection, these species can shed virulent AOAV-1 for extended periods, particularly via feces, often in the absence of overt clinical symptoms [48]. AOAV-1 is transmissible to susceptible poultry through direct contact [46,49]. However, serial passages of PPMV-1 can lead to the acquisition of adaptive mutations that enhance virulence [9], potentially through increased replication efficiency [50]. Notably, certain PPMV-1 isolates display inherent virulence in Galliformes, without requiring prior host adaptation [46]. Recent analysis of the complete fusion (*F*) gene confirmed interspecies transmission of class II AOAV-1 strains, notably between Columbiformes and Galliformes, as well as intercontinental dissemination [51,52]. Recent research has also demonstrated its ability to infect wildlife species, including a great spotted woodpecker in China [37], as well as the role of wild species in disease transmission, with sparrows, crows, and quails acting as potential carriers and transmitters of AOAV-1 [53]. Additionally, there is evidence of AOAV-1 infection in Bovidae (cattle and sheep), Mustelidae (mink), Cercetidae (hamster), Muridae (mice), Leporidae (rabbit), Camelidae (camel), Suidae (pig), Cercophithecidae (monkeys), and Hominidae (humans) [54]. In addition to causing general symptoms like anorexia, it can lead to respiratory infections in both humans and pigs, neurologic infections in monkeys and minks, and gastrointestinal infections in pigs [54].

Zoonotic transmission of AOAV-1 to humans is infrequent and generally results in transient conjunctivitis with no severe clinical outcomes, especially in individuals with close contact to infected birds. These individuals develop high antibody titers against AOAV-1, indicating immune exposure without significant disease. Nonetheless, more serious outcomes have been reported, such as flu-like illness, fatal pneumonia in immunocompromised patients [54], as well as a case of fatal meningoencephalitis in a 2-year-old recipient of hematopoietic stem-cell transplantation for combined immunodeficiency caused by PPMV-1 [52]. These findings highlight the potential for AOAV-1 to cause severe disease in immunocompromised individuals, emphasizing the need for vigilance in monitoring zoonotic transmission risks.

The extensive global spread, genetic diversification, and host adaptation of PPMV-1 (Genotype VI AOAV-1), as well as evidence of interspecies and intercontinental transmission, contribute to significant viral genetic variability. This ongoing evolution can result in mutations in key genomic regions targeted by standard molecular diagnostic tools. Consequently, current WHOA and USDA-recommended primer sets targeting the matrix (*M*) gene [1,10,12], designed based on earlier or reference sequences, may fail to detect certain variants, including the isolate characterized in our study [1,55,56]. Different, more sensitive primer sets have already been developed [36], focusing on different regions, such as the fusion (*F*) gene. Mutations in primer binding regions can reduce binding efficiency, leading to false negatives or decreased assay sensitivity, as observed in *AOAV-1*, where older primer sets failed to detect many circulating PPMV-1 strains, necessitating primer redesign [35]. Moreover, mutations at the F gene cleavage site and other evolving regions underscore the need for continuous monitoring and assay updates to ensure diagnostic accuracy. Similar challenges have been reported in other viral diagnostics, such as the mpox virus, where primer site mutations led to performance drops, highlighting the broader epidemiological risk posed by such mutations in rapidly evolving pathogens [52]. This highlights the critical need to continuously update and validate diagnostic assays to ensure accurate detection and surveillance of AOAV-1, particularly given its zoonotic potential and impact on diverse host species.

In Portugal, Newcastle disease is classified as a notifiable disease [57] and must be reported to the European Commission and the WHOA [1], as part of ongoing surveillance efforts. Furthermore, under the provisions of Edict No. 3 on Newcastle disease, dated 28 March 2019, vaccination against Newcastle disease is mandatory across the national territory for chickens, turkeys, and pigeons [58].

Genotype VI AOAV-1 (PPMV-1) presents a significant risk to domestic poultry, particularly commercial chickens, as well as wild bird populations. Effective control requires targeted interventions such as increasing awareness among pigeon owners and implementing strict biosecurity measures [36,59]. Although vaccines against AOAV-1 exist, their application in wild and feral bird populations is not feasible. Genotype VI viruses exhibit notable antigenic variation, and interspecies transmission from Columbiformes to Galliformes remains a concern [12,60,61]. Sustained monitoring of genotype VI is essential to understand its epidemiology and guide control strategies. Preventing contact between Columbiformes and poultry, especially around shared feed and water sources, is critical to minimizing cross-species transmission [62,63].

While the present study provides valuable insights, certain limitations must be acknowledged when interpreting the findings. The small number of pigeons examined, combined with the fact that many samples were unsuitable for histopathology or molecular analysis due to advanced autolysis, inevitably reduced the strength of the conclusions that could be drawn. Moreover, although the negative results of the M-gene PCR were later explained by the detection of a primer–sequence mismatch, this observation relied exclusively on in silico analysis. Without experimental validation, such as PCR using alternative primer sets, assays with synthetic oligonucleotides, or comparative analyses of Ct values and limits of detection, the impact of the mismatch on diagnostic sensitivity remains uncertain. The absence of further testing with F-gene-based or updated pan-AOAV primer sets represents an additional shortcoming that limited the robustness of the molecular confirmation. In light of the considerable genetic diversity of AOAV-1 variants circulating in the region, the broader epidemiological implications of mutations affecting primer binding sites also warrant deeper discussion than was possible here. For these reasons, the evidence presented supports the notion of a diagnostic limitation in standard M-gene PCR assays, but the conclusion that PCR failure can be definitively attributed to primer–sequence mismatch must remain cautious until reinforced by stronger experimental confirmation.

## 5. Conclusions

This study confirms the presence of the velogenic AOAV-1 Genotype VI (PPMV-1) in wild *Streptopelia decaocto* populations in our study area, highlighting wild Columbiformes as potential reservoirs and vectors of the virus. Metagenomic sequencing enabled the detection and partial characterization of these highly virulent viruses, revealing lesions consistent with velogenic infection. The failure of detection using the conventional M gene-based PCR assay was not due to PCR methodology itself but to limitations of the still-recommended M-gene primer set, which may miss genetically diverse AOAV-1 strains. These findings underscore the ongoing risk of PPMV-1 transmission to domestic poultry and, occasionally, to humans, reinforcing the importance of continued surveillance, updated diagnostic tools, strict biosecurity measures, and targeted awareness efforts to mitigate disease spread.

## Data Availability

The data presented in this study are available on request from the corresponding author.
